# Education and training in low-dose CT lung cancer screening across Europe: a survey study

**DOI:** 10.1186/s13244-026-02362-w

**Published:** 2026-07-29

**Authors:** Rebecca Mura, Pamela Zolda, Roberta Eufrasia Ledda, David R. Baldwin, Georgia Hardavella, Luis Seijo, Anna Kerpel-Fronius, Joanna Chorostowska-Wynimko, Torsten Gerriet Blum, Helmut Prosch

**Affiliations:** 1 Department of Biomedical Imaging and Image-guided Therapy, Medical University of Vienna, Vienna, Austria; 2 Department of Medicine and Surgery, University of Parma, Parma, Italy; 3 Respiratory Medicine, Nottingham University Hospitals NHS Trust, Nottingham, UK; 4 University of Nottingham, Nottingham, UK; 5 6th Department of Respiratory Medicine, “Sotiria” Athens’ Chest Diseases Hospital, Athens, Greece; 6 Department of Pneumology, Clínica Universidad de Navarra, Madrid, Spain; 7 Department of Radiology, National Korányi Institute for Pulmonology, Budapest, Hungary; 8 Department of Genetics and Clinical Immunology, National Institute of Tuberculosis and Lung Diseases, Warsaw, Poland; 9 Medical School Berlin, Berlin, Germany; 10 Department of Pneumology, Lungenklinik Heckeshorn, Helios Klinikum Emil von Behring, Berlin, Germany; 11 Christian Doppler Laboratory for Machine Learning Driven Precision Imaging, Department of Biomedical Imaging and Image-guided Therapy, Medical University of Vienna, Vienna, Austria

**Keywords:** Education, Lung cancer screening, SOLACE, Training

## Abstract

**Objectives:**

To explore the status of low-dose CT lung cancer screening (LCS) training practices, identify existing gaps, and define key competencies to be included in LCS educational curricula.

**Materials and methods:**

As part of the European SOLACE project, a structured cross-sectional survey consisting of 11 closed- and open-ended items, developed based on relevant literature, international guidelines, and expert input to assess LCS current practices and training needs, was administered to a panel of European LCS experts. Participants were invited to individual Zoom interviews (May–November 2025). Data were analyzed using descriptive statistics.

**Results:**

Twenty-five LCS experts were interviewed from 14 European countries, including 10 radiologists (40%), 8 pulmonologists (32%), 4 thoracic surgeons (16%), 1 project manager (4%), 1 smoking cessation specialist (4%), and 1 general practitioner (GP) (4%). Reported training activities ranged from established programmes (4/14 countries), to learning initiatives (6/14) and/or planned programmes (4/14). Radiologists (96%), pulmonologists (80%), and GPs (60%) were identified as the main target groups for training. On a 6-point Likert scale (0 = not important, 5 = extremely important), experts indicated limited awareness of training needs as the most relevant gap (mean 2.8 ± 2.2). Key educational areas to be strengthened included management of incidental findings (4.5 ± 0.6) and confident use of guidelines for nodule management (3.8 ± 1.8). Among essential competencies for professionals involved in LCS, the highest-rated were competence in managing incidental findings (4.7 ± 0.6), familiarity with guideline-based nodule management (4.7 ± 0.6), basic knowledge of AI tools (4.5 ± 0.8), and communication with LCS participants (4.5 ± 1.1).

**Conclusion:**

The findings suggest a heterogeneous LCS training landscape across European countries and underscore the importance of developing shared European curricula. Key priorities may include strengthening technical skills alongside structured communication training.

**Key Points:**

- Education and training of all stakeholders are essential for the successful implementation of LCS programmes.
- This survey assesses the current status of LCS training practices across Europe, identifies existing gaps, and defines key competencies to inform future curricula for health professionals involved in LCS.
- The European LCS training landscape is highly heterogeneous, with substantial variability in training activities, target professionals, and delivery modalities.

**Graphical Abstract:**

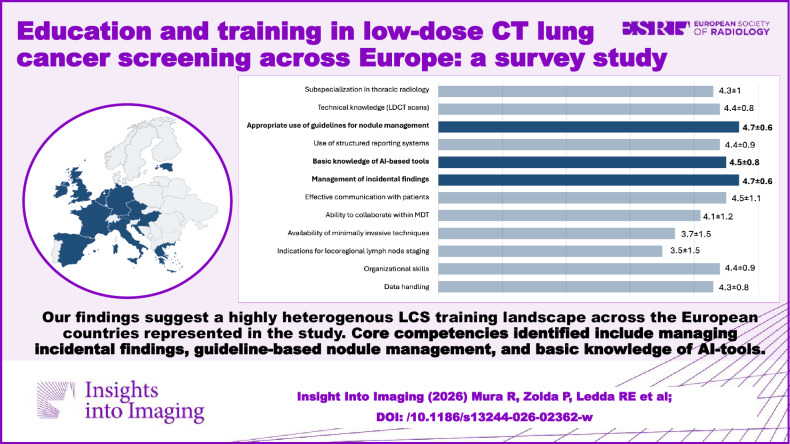

## Introduction

Lung cancer (LC) remains the leading cause of cancer-related mortality worldwide [1] and in Europe, where it accounts for about 20% of all cancer deaths [2]. Low-dose computed tomography (LDCT)-based lung cancer screening (LCS) enables the early detection of LC in high-risk individuals, resulting in a reduction of LC-specific mortality [3, 4]. Despite this robust evidence, nationwide implementation of LCS programmes across Europe remains limited [5–7]. The EU-funded “Strengthening the screening of Lung Cancer in Europe” (SOLACE) project aims to support and streamline the structured implementation of LCS programmes among EU member states [8]. However, to advance this process, several challenges still need to be addressed.

As outlined in the 10-pillar model of LCS proposed by Fintelman et al in 2015, the education of all stakeholders is essential for a successful screening program, recognizing that LCS requires specific knowledge and expertise [9, 10]. Similarly, the 2023–2027 Roadmap by the International Association for the Study of Lung Cancer (IASLC) highlights the importance of dedicated LCS training programmes to ensure appropriate interpretation of CT findings, optimize clinician workload, and ultimately benefit participant outcomes [11]. In Europe, a technical standard for comprehensive high-quality LCS was published in 2023 following a European Respiratory Society (ERS) Taskforce. This sets out the elements that are essential to a successful programme and includes details on initial training and downstream quality assurance [12].

Several international voluntary training initiatives have already been developed to support professional awareness and education in LCS, including programmes such as the European Society of Thoracic Imaging (ESTI) LCS Certification Project, the European School of Radiology (ESOR) LCS course series, and the educational activities promoted by the American College of Radiology (ACR) [13*–*15]. However, a comprehensive and standardized approach to multidisciplinary training across Europe has not yet been fully established.

Building on these considerations, the SOLACE project has dedicated a Work Package aimed at advancing the development of standardized training programmes for all professional groups involved in LCS [8]. This effort aims not only to accelerate the implementation of LCS across Europe but also to ensure its long-term quality and sustainability.

The objective of this survey study was to assess the current status of LCS training practices in Europe, identify existing gaps, and define key competencies that should be incorporated into future education and training curricula for health professionals engaged in LCS programmes.

## Materials and methods

This study followed three main phases: survey development, expert panel selection, and data collection through structured online interviews. The study did not include patient health data, therefore, formal ethics approval was not required.

### Survey design

A structured, cross-sectional survey conducted through individual interviews was designed to collect information on existing training practices related to LCS in Europe from an interdisciplinary panel of experts. The questionnaire, consisting of 11 items, was developed based on a review of the relevant literature, current international guidelines, and expert inputs. Items 1–10 included both closed- and open-ended questions, grouped in two main sections:

1. Status of LCS programmes and LCS education and training in each country
2. Personal insights and recommendations regarding LCS curricula

Item 11 was an optional open-ended question inviting any additional comments, helping ensure that no major themes relevant to the study objectives were missed.

In the second section, items 7–9 were assessed using a 6-point Likert scale (0 = not important, 5 = extremely important) to evaluate perceived gaps, areas requiring strengthening, and essential competencies. Additional comments submitted in this section under the “Other” category were first classified into thematic groups and then analyzed (see Supplementary Material_Table 1).

To ensure content clarity and relevance, the draft questionnaire was independently reviewed by three clinicians with expertise in LCS from different specialties (radiology, thoracic surgery, and pulmonology) who were not part of the final survey panel. These reviewers assessed the clarity, relevance, and completeness of the items, and minor revisions were made based on their feedback. Subsequently, the revised questionnaire underwent a pre-testing phase with an additional LCS expert (H.P.), who later participated in the survey, to evaluate overall clarity and feasibility, including the estimated completion time. Minor refinements were made before final administration. A copy of the final questionnaire is provided in the Supplementary Material.

### Expert panel selection

European experts were identified through professional networks, publications, and involvement in national and/or regional LCS projects and programmes, with part of the initial expert pool identified within the framework of the SOLACE project. In addition, a snowball approach was adopted, whereby some of the initially contacted experts recommended further eligible colleagues from their national networks. The aim was to assemble an expert panel that captured the diversity of perspectives on LCS training and education across different European contexts, reflecting the influence of diverse healthcare systems, and stages of screening implementation. Inclusion criteria required participants to have recognized expertise in one discipline related to LCS (e.g., radiology, pulmonology, thoracic surgery, etc). To ensure balanced geographical and professional representation while minimizing overrepresentation from any single project or country:

- A maximum of three LCS projects or programmes (either ongoing or completed) per country could be represented
- A maximum of one expert per discipline per country was included.

### Survey administration

A total of 35 experts were contacted via email using a standardized invitation, describing the purpose of the study and the voluntary nature of participation. Participants were then invited to schedule an individual Zoom interview. The interviews were conducted by a board-certified radiologist using a standardized script (R.M.), and sessions were designed to last approximately 30 min. This approach represents an interviewer-administered survey conducted through structured individual interviews, designed to ensure consistency in data collection across participants. All the interviews took place between May and November 2025. Interviews were audio-video recorded via Zoom, and transcripts were generated using an automated transcription software to support data review.

### Statistical analysis

Descriptive statistics were computed, and results were reported as counts and percentages. Likert-scale items were analyzed as interval data, as commonly done in survey research when response categories are assumed to be approximately equidistant; therefore, they were summarized using means and standard deviations (SD). Analyses were performed using IBM SPSS Statistics, Version 31.0 (IBM Corp., Armonk, NY, USA).

## Results

### Respondents’ characteristics

Twenty-five experts were interviewed (response rate 71.4%, 25/35) from 14 European countries. Participants included 10 radiologists (40%), 8 pulmonologists (32%), 4 thoracic surgeons (16%), 1 project manager (4%), 1 smoking cessation specialist (4%), and 1 general practitioner (GP) (4%) (Table 1A).

**Table 1:** Characteristics of survey respondents (*n* = 25)

A. Characteristics of survey respondents (*n* = 25)		
	Respondents (*N*)	%
Total	25	100%
Country		
Italy	2	8%
Austria	1	4%
Hungary	2	8%
Czech Republic	2	8%
Croatia	3	12%
Germany	2	8%
Spain	3	12%
France	1	4%
UK	1	4%
Belgium	2	8%
Netherlands	3	12%
Estonia	1	4%
Ireland	1	4%
Greece	1	4%
Profession		
Radiologist	10	40%
Pulmonologist	8	32%
Thoracic Surgeon	4	16%
GP	1	4%
Project manager	1	4%
Smoking cessation specialist	1	4%

B. Country-level LCS programme characteristics		
	*N*	%
Total	14	100%
Characteristics		
Countries with ongoing LCS programmes	14	100%
Nationwide established programmes	2	14.3%
Regional and/or pilot programmes	12	85.7%
Planned transition to nationwide programmes (≤ 5 years)	3	21.4%
Professionals involved in core teams		
Radiologists	14	100%
Pulmonologists	13	92.9%
GPs	12	85.7%
Thoracic surgeons	7	50%
Pathologists	3	21.4%
Ambassadors^*^	2	14.3%
Radiographers	10	71.4%
Smoking cessation specialist	8	57.1%
Programme coordinator	6	42.3%
Others	7	50%

^*^Ambassadors are trained community outreach personnel involved in engaging underserved populations and promoting participation in screening programmes, particularly among socially deprived groups and ethnic minorities

All represented countries (14/14, 100%) reported an ongoing LCS programme, but at different stages of implementation. Two countries (14.3%) had a nationwide established programme, while 12 (85.7%) were conducting regional programmes and/or pilot initiatives; among these, 3 countries (21.4%) planned to transition to nationwide implementation within the next 5 years.

Healthcare professionals involved in core screening teams varied, although radiologists (100%), pulmonologists (92.9%), and GPs (85.7%) were most frequently reported (Table 1B).

### LCS education and training characteristics

LCS education and training activities differed widely across participating countries. Established training programmes were identified in 4/14 (28.6%) countries, primarily targeting radiologists (4/4) and GPs (2/4), and in single cases, radiographers, nurses, and pulmonologists. Of these, two (the United Kingdom and France) were mandatory. Programme structure varied both by country and professional group. GP-oriented programmes were predominantly online and shorter in duration (approximately 30 min), whereas radiologists generally received hybrid training that included 1–3 days of practical sessions. One country (the United Kingdom) repeated its certification programme annually. Proof of activity (i.e., annual LDCT reading thresholds) was required for radiologists in 2/4 programmes, and a final examination was mandatory in 3/4 programmes (Table 2).

**Table 2:** Characteristics of training activities across participating countries (*n* = 14)

Training activity	Countries *n* (%)^*^	Main target professionals^*^	Mandatory yes/no; *n*	Assessment/certification (n)
Established programmes	4 (28.6%)	Radiologists (4/4) GPs (2/4) Radiographers (1/4) Pulmonologists (1/4) Nurses (1/4)	Yes, 2/4	Final exam (3/4), activity threshold (2/4)
Planned programmes	4 (28.6%)	Radiologists (4/4) GPs (2/4) Pulmonologists (2/4)	**-**	Not yet defined
Learning initiatives	6 (42.9%)	Radiologists	no	ESTI voluntary certification (4), internal QA (2)

^*^ Multiple responses were allowed for selected items

Planned programmes were identified in another 4/14 countries, also mainly directed at radiologists (4/4), GPs (2/4), and pulmonologists (2/4). For planned programmes, available information was more limited, as these initiatives are at an early stage of development. Only the format of training was reported consistently, ranging from online sessions to hybrid modules, and even a university-level program in the form of an approved master’s degree, planned to be delivered virtually through a university-based academic center (joint SEPAR-ALAT initiative in Spain) (Table 2).

Learning initiatives were more frequent, with 6/14 (42.9%) countries describing online (2/6) or hybrid (4/6) activities, typically addressed to radiologists. In addition, despite being internationally available, the ESTI  certification was mentioned as a voluntary certification only by respondents from 4 countries, while 2 countries described internal quality-assurance mechanisms, comprising activity-related criteria and coordinator-based evaluation of the healthcare professionals involved. Only one country (France) reported a legally mandated certification for radiologists, with qualification assessment performed by the national professional society. This certification pathway was described as based on the ESTI certification program and adapted at the national level. Another country (Germany) reported that training certification was expected to become mandatory with the transition to a national programme.

### Experts’ perspectives on LCS curricula

Experts indicated that training should primarily involve radiologists (24/25; 96%), pulmonologists (20/25; 80%), and GPs (15/25; 60%) (Table 3).

**Table 3:** Target audience of LCS education activities according to experts' recommendations

Specialty	*N* (%)
Total	25 (100%)
Radiologist	24 (96%)
Pulmonologist	20 (80%)
GP	15 (60%)
Thoracic surgeon	11 (44%)
Radiographer	8 (32%)
Program coordinator/manager	9 (36%)
Smoking cessation specialist	6 (24%)
Oncologist	2 (8%)
Radiotherapist	2 (8%)
Pathologist	2 (8%)

**Fig. 1:**
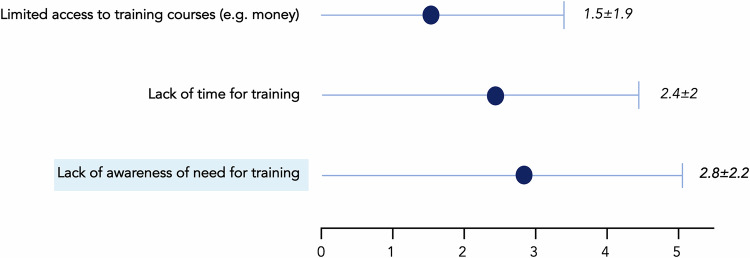
Expert ratings of proposed gaps in LCS training and education on a 6-point Likert scale (0 = not important, 5 = extremely important). Results are shown as mean ± SD

**Fig. 2:**
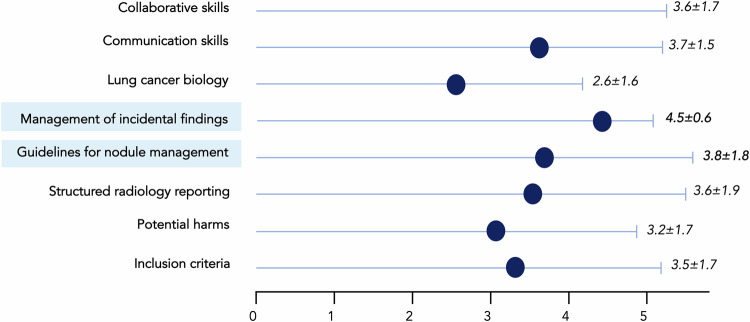
Expert ratings of key educational areas requiring strengthening in LCS training and education, using a 6-point Likert scale (0 = not important, 5 = extremely important). Results are shown as mean ± SD

**Fig. 3:**
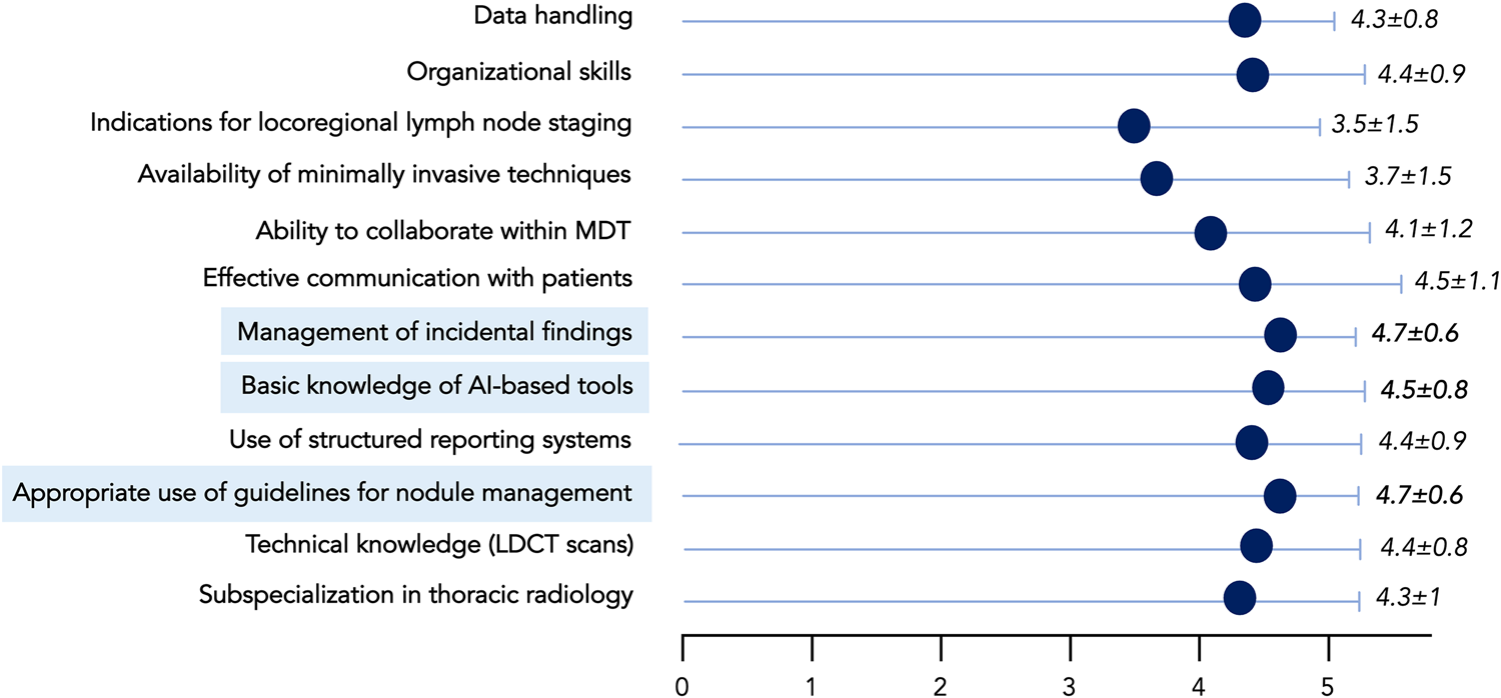
Expert ratings on essential competencies for professionals involved in LCS, using a 6-point Likert scale (0 = not important, 5 = extremely important). Results are shown as mean ± SD

For the following questions, one respondent indicated that the items were outside their area of expertise and therefore did not provide responses. On a 6-point Likert scale, experts rated *limited awareness of training needs* (mean 2.8 ± 2.2) as the most relevant gap among those proposed. Open responses provided under the “Other” category most frequently mentioned *LCS awareness and perceived needs* (5/24) (Fig. 1). Key educational areas to be strengthened included *management of incidental findings* (mean 4.5 ± 0.6) and *confident use of guidelines for nodule management* (mean 3.8 ± 1.8) (Fig. 2), while respondents additionally highlighted *smoking cessation* (4/24) and *downstream clinical workflow following CT scans* (4/24). Regarding essential competencies for professionals involved in LCS, the top-rated were *competence in managing incidental findings* (mean 4.7 ± 0.6), *familiarity with guideline-based nodule management* (mean 4.7 ± 0.6), *basic knowledge of AI-based tools* (mean 4.5 ± 0.8), and *effective communication with LCS participants* (mean 4.5 ± 1.1). *Awareness and education regarding smoking cessation* emerged most frequently among additional responses (3/24) (Fig. 3).

Most experts (23/24 respondents, 95.8%) supported integrating LCS-related education into residency curricula, particularly in radiology (22/23, 95.7%), pulmonology (13/23, 56.5%), thoracic surgery (12/23, 52.2%), and general practice (10/23, 43.5%). Among “Other” responses, oncologists were most frequently suggested (5/8). Concerning the optimal timing of training, 10/23 (43.5%) respondents indicated the senior years of residency, while 13/23 (56.5%) found the timing either not relevant or difficult to define. Similarly, most respondents found it challenging to estimate the duration of training (defined as the total training time that residents should dedicate to LCS activities), as the structure and requirements of residency programmes may vary substantially across specialities and countries. Among the four (17.4%) who provided an estimate, suggestions for a basic LCS training module ranged from one week to two months. Two participants (8.7%) also recommended introducing LCS awareness in medical school, and one (4.3%) recommended extending awareness to all medical specialties. Finally, 21 out of 24 respondents (85.7%) supported resident involvement in multidisciplinary team (MDT) discussions (Table 4).

**Table 4:** Experts’ views on integration of LCS-related education into residency curricula

Item	n/N (%)
Support integration of LCS education into residency curricula	23/24 (95.8%)
Support resident involvement in MDT discussions	21/24 (87.5%)
Which specialties should undergo an LCS training?^*,**^	
Radiology	22/23 (95.7%)
Pulmonology	13/23 (56.5%)
Thoracic surgery	12/23 (52.2%)
General practice	10/23 (43.5%)
Others	8/23 (34.8%)
Oncology	5/8 (62.5%)
Other suggestions^*^	
Introduce LCS awareness in medical school	2/23 (8.7%)
Extend LCS awareness to all medical specialties	1/23 (4.3%)

^*^ Only respondents who supported the integration of LCS education into residency curricula were included

^**^ Multiple responses were allowed for selected items

Responses to the 11th open-ended item were highly heterogeneous and not suitable for systematic categorization. Review of these comments did not identify additional themes beyond those already addressed by the structured items; rather, they reinforced previously identified priorities such as awareness of LCS, quality assurance, smoking cessation training, and the need for harmonized European training frameworks. Therefore, these responses were not included in the formal analysis.

## Discussion

This survey, conducted within the SOLACE project and involving European experts from various LCS-related disciplines, provides an overview of current practices and perceptions related to education and training in this field.

The first observation emerging from the data is the substantial heterogeneity of LCS-related training across Europe, which remains largely non-standardized and, in many settings, only partially developed. Despite the importance of educating all stakeholders to ensure effective LCS implementation, which has long been recognized [9, 12], only 4 out of the 14 participating EU countries reported having an established educational programme. Moreover, the target audiences of these programmes were not fully harmonized. In fact, while all four programmes addressed radiologists, the involvement of other professional groups (GPs, radiographers, nurses, pulmonologists) differed considerably. Delivery formats were also highly variable, ranging from short, fully online courses to hybrid models that include practical sessions. Further variability concerns the evaluation methods: not all programmes request a final examination (reported in 3/4 cases), and proof-of-activity requirements for radiologists (i.e., annual LDCT reading thresholds) were also inconsistent (documented in 2/4 cases). Furthermore, a few programmes addressed the need for ongoing education and quality assurance mechanisms. In this regard, continuing medical education, certification, and recertification processes represent important components of sustainable and high-quality screening programmes. Although these aspects were not explored in detail within the present survey, they deserve further consideration in the development of future LCS educational frameworks.

Together, these discrepancies underscore a clear gap between the widely recognized need for training and the current limited availability of standardized learning opportunities. In addition, although the voluntary ESTI certification represents a structured and accessible tool to support radiologists’ quality assessment, its diffusion appears limited. Indeed, the ESTI certification was mentioned as an available voluntary option in only 4 out of 14 countries. This aligns with official data showing that, between 2022 and 2025, only 77 European radiologists obtained the certification [13]. This relatively modest uptake suggests that, despite the availability of an internationally recognized training opportunity, its implementation across EU LCS settings remains far from widespread. This probably reflects structural, organizational, and cultural barriers that make it difficult for radiologists across Europe to pursue a specialized certification, even when it is high‑quality and internationally recognized. Established workforce shortages, clinical workload pressures, competing educational priorities, lack of institutional/national mandates, as well as limited educational reimbursement for training courses may be a few of the reasons explaining this low uptake.

With regard to radiologists, useful reference models for developing LCS training strategies come from countries with established LCS systems. The United Kingdom, which currently hosts the largest national program in Europe, has implemented PERFECTS, a web-based external quality-assurance system modeled after the national health system (NHS) breast screening programme [16, 17]. It evaluates, on an annual basis, readers’ ability to detect and interpret nodules, provides benchmarking against national performance standards, and identifies those requiring additional training. Similarly, the British Columbia Lung Screening Program in Canada requires radiologists to complete structured training modules covering key areas such as nodule classification, nodule-measurement methods, and management of incidental findings, complemented by practical sessions on computer-assisted reporting [18]. Maintenance of certification requires reading at least 500 LDCT examinations per year [18]. Notably, the Canadian Program also defines mandatory training standards for pulmonologists and thoracic surgeons [18].

Regarding which specialities should receive LCS-related training, the experts unsurprisingly identified radiologists, pulmonologists, and GPs as the primary target groups, reflecting their central roles in most programmes. Notably, a non-negligible proportion of respondents (24%) also indicated smoking cessation specialists, underscoring that LCS represents not only a secondary prevention strategy but also a teachable moment to promote smoking-cessation interventions—a combination shown to maximize reductions in LC-specific and overall tobacco-related mortality [19, 20]. This finding further highlights the role of LCS as a critical opportunity for primary prevention and underscores the need for smoking cessation competencies to be integrated into training programmes for all LCS stakeholders and systematically implemented within screening pathways. Experts also agreed that LCS education should ideally be introduced during residency for the disciplines involved in screening. However, several respondents cautioned that, while early integration is desirable, it may still be premature to decide on this at the current stage of LCS development in Europe.

Among the obstacles to the implementation of structured LCS training programmes, the most frequently reported was limited awareness of the need for dedicated training, suggesting that the value of LCS-specific competencies is not yet fully recognized. Other potential barriers—such as lack of time and/or financial constraints—received considerably lower ratings. This pattern may suggest that, if LCS training becomes mandatory, stakeholders expect institutional or health-system support, including protected time or financial coverage. Open-ended responses highlighted two additional themes: motivation and institutional/political support. Several experts referred to a general lack of incentives to participate in LCS training, noting that the importance of LCS is insufficiently promoted, and health professionals often perceive limited financial or developmental motivations to pursue training in this area. These observations suggest that, beyond structural barriers, professional engagement and institutional prioritization may influence training uptake. This data can also potentially explain the limited number of radiologists being certified by the dedicated ESTI training course [13], therefore highlighting important factors to be considered moving forward in establishing national LCS training programmes. Interestingly, despite the well-documented shortage of dedicated chest radiologists across Europe [5], respondents did not identify this as a barrier to implementing structured LCS training programmes.

Conversely, it is not surprising that among key educational areas to be strengthened, management of incidental findings received the highest rating (mean 4.5), well above guideline-based nodule management (mean 3.8). This result likely reflects not only the limited availability of dedicated training but also a broader lack of standardization across Europe regarding what to report and how to manage these findings within LCS [21, 22], despite a multi-European society consensus statement published in 2023 [23]. However, the management of incidental findings in LCS (e.g., coronary artery calcification, chronic obstructive pulmonary disease (COPD)) is anything but trivial. Unlike established cancer screening programmes (such as breast or colorectal screening), which are single-organ and cancer-centered, LCS holds a multi-organ and multi-disease prevention potential. Consequently, it requires training that goes beyond established screening practices for other cancers, extending a patient-centered management of incidental findings and their associated risk factors and lifestyle choices. This inherent complexity represents both a challenge and an opportunity and underscores the need for education and training to be planned from the outset, to ensure and sustain high-quality screening standards.

Communication skills also emerged as an area needing improvement (mean 3.7). Effective communication and shared or “informed” decision-making represent a critical component in LCS, as they can improve adherence to screening and follow-up recommendations [24]. Although communication with participants may often rely on referring physicians, the significant role of radiologists in LCS offers them an opportunity to “come out of the dark”, meet, and educate participants, reinforcing the need for dedicated communication training [24, 25].

Regarding essential competencies, experts prioritized management of incidental findings, guideline-based nodule management, and basic knowledge of AI-based tools. These areas reflect major clinical priorities, as nodule management remains the cornerstone of LDCT-LCS, while the growing emphasis on familiarity with AI systems is in line with the increasing integration of computer-aided detection (CAD) and automated volumetric assessment into the LCS workflow, where AI-based tools are already used as concurrent or second readers [26].

This study has several limitations. First, the sample size was relatively small, and the radiologists were slightly over-represented compared with other professional groups, potentially influencing the prioritization of competencies. Second, geographic participation was not fully representative, with limited involvement from Northern and Eastern European countries, which may limit the generalizability of the findings. Third, the predefined items inevitably reflect the authors’ perspective, and the survey was administered in English, which may have influenced item interpretation for some respondents. In addition, as participation was voluntary and recruitment was also based on professional networks and snowball sampling, the possibility of selection bias cannot be excluded. Finally, as with all self-reported survey data, the findings reflect participants’ opinions and perceived training needs rather than objective measurements of training quality and may therefore be subject to reporting bias.

### Future perspectives

Future steps should focus on translating these findings into an actionable framework for harmonized LCS education and training across Europe. This would include the development of shared EU-level core curricula and minimum competency standards adaptable to different national contexts.

## Conclusion

The findings suggest a highly heterogeneous LCS training landscape across the European countries represented in the study, further challenged by limited awareness of training needs, workforce limitations and logistical constraints, and highlight the importance of developing shared European curricula. Priorities also include defining common target groups and harmonizing training delivery modalities.

Key competencies identified include technical skills such as the management of incidental findings, guideline-based nodule management, and basic knowledge of AI-based tools—areas that align with current clinical priorities and ongoing integration of AI into LCS practice—alongside structured communication training.

These elements could represent a starting point for the further development of standardized and pan-European educational curricula that may support a consistent and high-quality training pathway for health professionals involved in LCS programmes.

## Supplementary information

ELECTRONIC SUPPLEMENTARY MATERIAL

## Data Availability

The datasets used and analyzed during the current study are available from the corresponding author on reasonable request.

## Abbreviations

ACR: American College of Radiology
ESOR: European School of Radiology
ESTI: European Society of Thoracic Imaging
ERS: European Respiratory Society
GP: General practitioner
LDCT: Low-dose computed tomography
LC: Lung cancer
LCS: Lung cancer screening
SOLACE: Strengthening the screening of Lung Cancer in Europe

## References

[1] Bray F, Laversanne M, Sung H et al (2024) Global cancer statistics 2022: GLOBOCAN estimates of incidence and mortality worldwide for 36 cancers in 185 countries. CA A Cancer J Clin 74:229–263. 10.3322/caac.21834 10.3322/caac.21834 38572751

[2] Malvezzi M, Santucci C, Boffetta P et al (2023) European cancer mortality predictions for the year 2023 with focus on lung cancer. Ann Oncol 34:410–419. 10.1016/j.annonc.2023.01.010 36882139 10.1016/j.annonc.2023.01.010

[3] De Koning HJ, Van Der Aalst CM, De Jong PA et al (2020) Reduced lung-cancer mortality with volume CT screening in a randomized trial. N Engl J Med 382:503–513. 10.1056/nejmoa1911793 31995683 10.1056/NEJMoa1911793

[4] The National Lung Screening Trial Research Team (2011) Reduced lung-cancer mortality with low-dose computed tomographic screening. N Engl J Med 365:395–409. 10.1056/nejmoa1102873 21714641 10.1056/NEJMoa1102873 PMC4356534

[5] Hardavella G, Frille A, Sreter KB et al (2024) Lung cancer screening: Where do we stand? Breathe 20:230190. 10.1183/20734735.0190-2023 39193459 10.1183/20734735.0190-2023 PMC11348916

[6] Kauczor H-U, Baird A-M, Blum TG et al (2020) ESR/ERS statement paper on lung cancer screening. Eur Respir J 55:1900506. 10.1183/13993003.00506-2019 32051182 10.1183/13993003.00506-2019

[7] Kauczor H-U, Bonomo L, Gaga M et al (2015) ESR/ERS white paper on lung cancer screening. Eur Respir J 46:28–39. 10.1183/09031936.00033015 25929956 10.1183/09031936.00033015 PMC4486375

[8] Kauczor H-U, Von Stackelberg O, Nischwitz E et al (2024) Strengthening lung cancer screening in Europe: fostering participation, improving outcomes, and addressing health inequalities through collaborative initiatives in the SOLACE consortium. Insights Imaging. 10.1186/s13244-024-01814-5 10.1186/s13244-024-01814-5 PMC11496428 39436577

[9] Fintelmann FJ, Bernheim A, Digumarthy SR et al (2015) The 10 pillars of lung cancer screening: rationale and logistics of a lung cancer screening program. Radiographics 35:1893–1908. 10.1148/rg.2015150079 26495797 10.1148/rg.2015150079

[10] Adams SJ, Flores EJ, Little BP et al (2024) *Radiographics* update: the 10 pillars of lung cancer screening—rationale and logistics of a lung cancer screening program. Radiographics. 10.1148/rg.230057 10.1148/rg.230057 PMC10878164 38329900

[11] Lam S, Bai C, Baldwin DR et al (2024) Current and future perspectives on computed tomography screening for lung cancer: a roadmap from 2023 to 2027 from the International Association for the Study of Lung Cancer. J Thorac Oncol 19:36–51. 10.1016/j.jtho.2023.07.019 37487906 10.1016/j.jtho.2023.07.019 PMC11253723

[12] Baldwin DR, O’Dowd EL, Tietzova I et al (2023) Developing a pan-European technical standard for a comprehensive high-quality lung cancer computed tomography screening programme: an ERS technical standard. Eur Respir J 61:2300128. 10.1183/13993003.00128-2023 37202154 10.1183/13993003.00128-2023

[13] ESTI (2026) ESTI lung cancer screening certification project_ESTI LCS holders n.d. https://www.myesti.org/lungcancerscreeningcertificationproject/. Accessed 10 Dec 2026

[14] ESOR (2026) ESOR lung cancer screening course n.d. https://www.esor.org/courses/special-focus/lung-cancer-screening-2nd-edition/. Accessed 13 Apr 2026

[15] ACR (2026) ACR lung cancer screening education. https://www.acr.org/Education-and-CME/Cancer-Screening-and-Staging/Lung-Cancer-Screening-Education. Accessed 13 Apr 2026

[16] NHS (2025) NHS_Quality assurance standards prepared for the targeted lung health checks programme. https://www.england.nhs.uk/wp-content/uploads/2019/02/PRN01867ii-quality-assurance-standards-prepared-for-the-lung-cancer-screening-programme-v3.pdf. Accessed 9 Dec 2025

[17] NHS (2025) NHS_Standard protocol prepared for the Lung Cancer Screening Programme. https://www.england.nhs.uk/wp-content/uploads/2019/02/PRN01867i-standard-protocol-prepared-for-the-lung-cancer-screening-programme-v3.pdf. Accessed 9 Dec 2025

[18] BC Cancer (2025) BC cancer lung screening standards and protocols. http://www.bccancer.bc.ca/screening/Documents/Lung-Screening-Standards-Protocols.pdf. Accessed 9 Dec 2025

[19] Cadham CJ, Jayasekera JC, Advani SM et al (2019) Smoking cessation interventions for potential use in the lung cancer screening setting: a systematic review and meta-analysis. Lung Cancer 135:205–216. 10.1016/j.lungcan.2019.06.024 31446996 10.1016/j.lungcan.2019.06.024 PMC6739236

[20] Meza R, Cao P, Jeon J et al (2022) Impact of joint lung cancer screening and cessation interventions under the new recommendations of the U.S. preventive services task force. J Thorac Oncol 17:160–166. 10.1016/j.jtho.2021.09.011 34648947 10.1016/j.jtho.2021.09.011 PMC8692396

[21] Ledda RE, Milanese G, Revel M-P, Snoeckx A (2025) Pros and cons of reporting incidental findings in lung cancer screening. Eur Radiol. 10.1007/s00330-025-11580-7 10.1007/s00330-025-11580-7 PMC12417295 40234338

[22] Henderson LM, Kim RY, Tanner NT et al (2025) Lung cancer screening and incidental findings: a research agenda: an official American Thoracic Society Research Statement. Am J Respir Crit Care Med 211:436–451. 10.1164/rccm.202501-0011ST 39928329 10.1164/rccm.202501-0011ST PMC11936151

[23] O’Dowd EL, Tietzova I, Bartlett E et al (2023) ERS/ESTS/ESTRO/ESR/ESTI/EFOMP statement on management of incidental findings from low dose CT screening for lung cancer. Eur Respir J 62:2300533. 10.1183/13993003.00533-2023 37802631 10.1183/13993003.00533-2023

[24] Snoeckx A, Franck C, Silva M, Prokop M, Schaefer-Prokop C, Revel M-P (2021) The radiologist’s role in lung cancer screening. Transl Lung Cancer Res 10:2356–2367. 10.21037/tlcr-20-924 34164283 10.21037/tlcr-20-924 PMC8182709

[25] Green DB, Pua BB, Crawford CB et al (2018) Screening for lung cancer: communicating with patients. AJR Am J Roentgenol 210:497–502. 10.2214/AJR.17.18836 29166146 10.2214/AJR.17.18836

[26] Geppert J, Asgharzadeh A, Brown A et al (2024) Software using artificial intelligence for nodule and cancer detection in CT lung cancer screening: systematic review of test accuracy studies. Thorax 79:1040–1049. 10.1136/thorax-2024-221662 39322406 10.1136/thorax-2024-221662 PMC11503082

