# Supplementary material for: Education and training in low-dose CT lung cancer screening across Europe: a survey study
Source: Insights Imaging. 2026 Jul 29;17:196. doi: 10.1186/s13244-026-02362-w (PMC13421576; doi:10.1186/s13244-026-02362-w)
Supplement: Supplementary file 1 — ELECTRONIC SUPPLEMENTARY MATERIAL [file 13244_2026_2362_MOESM1_ESM.pdf]

Education and Training in Low-Dose CT Lung Cancer Screening Across Europe: A Survey Study

ELECTRONIC SUPPLEMENTARY MATERIAL

Supplementary Table 1

Survey Item	Response	Thematic Categories
**Item 7** – Additional responses ("Other" option)		
	LCS awareness, which affect both utility and resources availability)	Awareness and perceived needs
	0	
	0	
	0	
	The lack of perceived need from the community of radiologists to be trained for LCS	Awareness and perceived needs
	Expand the training to other professionals, the lack of practical part integrated into the program	Training Structure and Availability
	The importance of LCS is not sufficiently promoted or incentivized, and there are no additional professional or financial motivations for young radiologists to pursue training in this area	Motivation and Incentives
	People think that they don't need specific education for doing LCS	Awareness and perceived needs
	Lack of visibility for the training program	Institutional and Political Support
	Lack of official support	Institutional and Political Support
	Homogenise how to read the LDCTs (different software)	Technical Issues
	Political will and pharmaceutical companies' support to implement LCS program development and consequently education	Institutional and Political Support
	Not enough slots for the workshops (necessary if you have to speed up the process)	Training Structure and Availability
	Radiology misconception of how current skills apply to LCS (e.g., excellent thoracic radiologists perform rather badly on the external quality assurance package before training)	Awareness and perceived needs
	0	
	0	
	Availability of courses	Training Structure and Availability
	0	
	Quality assurance metrics perceptions	Others
	Financial motivation for recruitment	Motivation and Incentives
	0	

	Smoking cessation (indicating gaps in awareness among referring physicians about the importance of smoking cessation as a core component of LCS)	Others
	0	
	0	
	Lack of motivation	Motivation and Incentives
**Item 8** – Additional responses ("Other" option)		
	0	
	0	
	0	
	0	
	Using of AI tools	AI tools
	Nodule management after being identified on CT, benefits of LCS	Nodule management after CT and further clinical Workflow
	Optimal implementation of AI tools	AI tools
	Smoking cessation program	Smoking Cessation
	Workflow of the system (not yet well-established)	Nodule management after CT and further clinical Workflow
	Training in logistics and operational issues	Organizational Aspects
	0	
	Lung nodules MDT	Nodule management after CT and further clinical Workflow
	0	
	Administrative Management	Organizational Aspects
	Stricter adherence to guidelines	Nodule management after CT and further clinical Workflow
	0	
	Different communication skills for reaching minorities	Patient care
	Financial aspects	Others
	Quality assurance metrics (all the processes, mainly radiology)	Others
	0	
	-	-
	0	
	Smoking cessation	Smoking Cessation
	Tobacco cessation counselling	Smoking Cessation
	Primary prevention (smoking cessation)	Smoking Cessation
**Item 9** – Additional responses ("Other" option)		

	0	
	0	
	0	
	0	
	0	
	0	
	Awareness and education regarding pharmacological support for smoking cessation	Awareness and education regarding pharmacological support for smoking cessation
	Ability to promote LCS (importance of dissemination and media)	Ability to promote LCS
	0	
	0	
	0	
	0	
	0	
	Management, infrastructure and automation	Management, infrastructure and automation
	Knowledge of epidemiological principles of the screening	Knowledge of epidemiological principles of the screening
	0	
	0	
	0	
	Dedicating time in the normal clinical workplan	Others
	Smoking cessation	Awareness and education regarding pharmacological support for smoking cessation
	-	-
	0	
	0	
	Tobacco cessation counselling	Awareness and education regarding pharmacological support for smoking cessation
	0	

Supplementary Table 2. Ratings of survey items related to training gaps, training areas to be strengthened, and competencies in lung cancer screening.
Ratings were provided on a Likert scale from 0 (not important) to 5 (extremely important). Values represent the number of respondents selecting each rating.
N/A indicates missing responses. Total N = 25 respondents.

Domain	Survey Item	5	4	3	2	1	0	N/A	Mean	SD	Median	IQR
**Training gaps and barriers**	Lack of awareness of need of training	10	3	2	0	3	7	-	2.8	2.2	4	0-5
**Training gaps and barriers**	Lack of time for training	5	5	3	3	0	9	-	2.4	2	3	0-4
**Training gaps and barriers**	Limited access to training courses (e.g. money)	3	1	5	1	2	13	-	1.5	1.9	0	0-3
**Areas that need to be strengthened**	Knowledge regarding inclusion criteria	9	6	3	2	1	3	1	3.5	1.7	4	2.25-5
**Areas that need to be strengthened**	Awareness of potential harms	6	7	5	1	2	3	1	3.2	1.7	4	2.25-4.75
**Areas that need to be strengthened**	Structured Radiology reporting	12	5	2	0	1	4	1	3.6	1.9	4.5	3-5
**Areas that need to be strengthened**	Confident use of guidelines for nodule management	14	3	2	1	1	3	1	3.8	1.8	5	3-5
**Areas that need to be strengthened**	Management of incidental findings	13	10	1	0	0	0	1	4.5	0.6	5	4-5
**Areas that need to be strengthened**	Basic knowledge of lung cancer biology	3	4	8	1	5	3	1	2.6	1.6	3	1-4
**Areas that need to be strengthened**	Communication skills	10	5	4	3	0	2	1	3.7	1.5	4	3-5
**Areas that need to be strengthened**	Collaborative skills	11	3	5	1	2	2	1	3.6	1.7	4	3-5
**LCS Competencies**	Sub-specialization in thoracic radiology	14	3	6	1	0	0	1	4.3	1	5	3-5
**LCS Competencies**	Technical knowledge to perform and interpret LDCT	15	4	5	0	0	0	1	4.4	0.8	5	4-5
**LCS Competencies**	Familiarity with and appropriate use of guidelines for nodule management	17	6	1	0	0	0	1	4.7	0.6	5	4-5
**LCS Competencies**	Ability to use structured reporting systems	14	6	3	1	0	0	1	4.4	0.9	5	4-5
**LCS Competencies**	Basic knowledge and application of AI-based tools	16	4	4	0	0	0	1	4.5	0.8	5	4-5

**LCS Competencies**	Competence in managing incidental findings	17	6	1	0	0	0	1	4.7	0.6	5	4-5
**LCS Competencies**	Effective communication with participants	16	7	0	0	0	1	1	4.5	1.1	5	4-5
**LCS Competencies**	Ability to engage and collaborate within a multidisciplinary team	12	6	5	0	0	1	1	4.1	1.2	4.5	3.25-5
**LCS Competencies**	Availability of minimally invasive techniques for diagnosis and treatment	10	4	6	1	2	1	1	3.7	1.5	4	3-5
**LCS Competencies**	Indications for locoregional lymph node staging in non-metastatic NSCLC	8	4	7	2	2	1	1	3.5	1.5	3.5	3-5
**LCS Competencies**	Organizational skills to manage workflows and coordinate LCS activities	15	5	3	1	0	0	1	4.4	0.9	5	4-5
**LCS Competencies**	Data handling	11	9	4	0	0	0	1	4.3	0.8	4	4-5

Survey about training & education in lung cancer screening

<u>Part I: Overview of the current status of lung cancer screening (LCS) training in your country</u>

1. **Are there any ongoing or planned LCS programs in your country?**

- *Are these programs regional/pilot projects or nationwide programs?*
- *Duration?*

2. **Which healthcare professionals are involved in your LCS program?**

e.g. Primary Care Provider (GP, Pulmonologist), Radiologist, Radiographer, Thoracic Surgeon, Pathologist Program Coordinator, Tobacco Cessation Counsellor, Ambassador

3. **Is there any ongoing training for healthcare professionals involved in LCS in your country?**

If yes:

- *Which specialties is it targeted to?*
- *Is it mandatory?*

If no:

- *Are you aware of any plans to develop such training?*

4. **If yes to question 3, How are training programs for healthcare professionals involved in LCS structured in your country?**

- *Which educational resources are used (e.g., classroom training, online courses, practical simulations)?*
- *Are they offered in different formats (e.g., in-person, online, hybrid)?*
- *How long do these training programs last?*
- *Do they need to pass a final examination?*

<u>Feel free to share any relevant official websites or resources you refer to.</u>

5. **Do healthcare professionals involved in LCS legally need any specific certification in your country?**

If yes:

- *For which specialties?*
- *Who assesses these qualifications (e.g., national/EU authorities or professional societies)?*
- *Is the certification program iteratively repeated (e.g. yearly, every 3 years, other) to warrant consistency and update for the LCS practice?*
- *Does the certification program include a proof of activity (e.g. a minimum number of LDCT reporting per year) to support its longitudinal maintenance?*

If no: ***Any other relevant and/or voluntary certification? (Same questions or official websites you refer to).***

Part II: Personal view and experiences

6. **In your opinion, which specialties should be trained?**

7. **Have you identified any specific training gaps or barriers to the implementation of the training program? Rate on a scale from 0 (not important) to 5 (extremely important).**

   - lack of awareness about the need for training
   - lack of time for training
   - limited access to training courses (time, money, etc)
   - Other (Please specify…):

8. **In your opinion, which areas of training should be strengthened to enhance the development and effectiveness of LCS programs in your field? Rate on a scale from 0 (not important) to 5 (extremely important).**

   - Knowledge regarding inclusion criteria
   - Awareness of potential harms
   - Structured radiology reporting
   - Confident use of guidelines for nodule management
   - Management of incidental findings
   - Basic knowledge of lung cancer biology
   - Communication skills (e.g., with patients, discussing results, shared decision-making)
   - Collaborative skills (e.g., teamwork in a multidisciplinary setting)
   - Other (Please specify…):

9. **In your opinion, how important are the following competencies and skills for the medical discipline primarily responsible for this task within an LCS program? Rate on a scale from 0 (not important) to 5 (extremely important).**

   - Sub-specialization in thoracic radiology
   - Technical knowledge to perform and interpret low-dose CT scans
   - Familiarity with and appropriate use of guidelines for nodule management
   - Ability to use structured reporting systems
   - Basic knowledge and application of AI-based tools
   - Competence in managing incidental findings
   - Effective communication with patients (e.g., explaining risk, results, limitations)
   - Ability to engage and collaborate within a multidisciplinary team
   - Availability of minimally invasive techniques for diagnosis and treatment
   - Indications for locoregional lymph node staging in patients with non-metastatic NSCLC

- Organizational skills to manage patient workflows and coordinate LCS activities
- Data handling and management skills (e.g., extracting relevant clinical information, etc)
- Other (Please specify…):

10. Do you think that LCS-related education should be integrated into the residency curriculum?

If yes:

- *For which specialties?*
- *When should this training be provided?*
- *And to what extent?*
- *Should residents be involved in Multidisciplinary Team discussions?*

11. Do you have any additional suggestions or comments regarding the training and education in LCS?
